# Mercury

**Published:** 1884-05

**Authors:** J. S. Todd

**Affiliations:** Professor Materia-Medica and Therapeutics in the Atlanta Medical College, Atlanta, Ga.


THE ATLANTA
MEDICAL AND SURGICAL JOURNAL.

VOL. I. MAY, 1884. No. 3.

### Original Communications.

### MERCURY.*

BY J. S. TODD, M. D.,

Professor Materia-Medica and Therapeutics in the Atlanta Medical College, Atlanta, Ga.

The first lecture was devoted to an exhibition of the various preparations of the metal, their physical and chemical properties, tests, doses, antidotes and history. Mercury has been known to science from the remotest antiquity. Aristotle, who flourished and wrote four centuries before the Christian era, mentions it. Paracelsus, the father of chemistry, first made corrosive sublimate, and thought in it he had discovered the Liquor Vitæ. He died a martyr to his faith. No drug has been so misused and abused; no other medicine so vaunted and so deprecated; no agent so unanimously commended by one school, and so universally condemned by another; no single one of all the various things and substances used for the cure of disease can claim as many victories over it as mercury; no article used for healing has filled as many premature graves; it is potential with good, but also capable of doing great harm; it is a two-edged sword, wielded skillfully, killing disease; ignorantly, the patient; it is a roaring lion, a huge Leviathan, a veritable Samson. Samson slew a thousand Philistines with the jawbone of an ass; in the hands of asses, calomel has killed a number like unto the sands on the seashore. By the wagging of the jaws of asses against

*A synopsis of four lectures from notes taken by L. B. V. Woolley and E. N. Shaw, students in attendance, session '83-84. Revised and corrected by the Professor.

its use, multitudes, for the lack of it, have died equal unto the stars in the firmament. Byron says of Corinth:

> "Many a vanished year and age,
> And tempest breath and battle rage,
> Has swept o'er Corinth, yet she stands
> A fortress formed by freemen's hands;
> The tempest's blast, the earthquake's shock,
> Has left untouched this hoary rock."

So of mercury, gentlemen, and the length of time it has claimed recognition as a potential agent against disease, has not only hallowed it, but has firmly established it; the electricity generated by the "tempest's breath" has purified the atmosphere, so to speak, and we see now clearly that which before was viewed dimly, hence not intelligently; the battles fought over it have killed off errors on both sides of the question; the shaking up which we were given by Hahnemann and the Eclectics, has caused us to abandon houses builded on the sand, and to-day, firmly established we hope on the solid rock of truth, battling against disease, sickness, suffering and death, I am verily persuaded that the rational uses of mercury are probably more pregnant with good to mankind than any other drug in our *armentarum medicorum.* When opium was in its swaddling clothes mercury had been known for two thousand years. Quinine is a thing of yesterday. It is one—alas! too few—of the drugs capable of entering the blood, grappling with disease and coming off the victor.

### PHYSIOLOGICAL ACTION.

The fumes of mercury coming in contact with seeds prevent their germination. A solution of corr. sub., 2 parts to the 1,000, destroys the communicability of vaccine virus; applied to roots of plants in soluble form it kills them. It destroys the embryo in the eggs of insects; water contaminated with it is poisonous to the fish and infusoria which live in it. In the neighborhood of furnaces where it is smelted cows abort and other animals lose flesh, become cachectic and frequently die. Men who work in mercury mines, smelt ores containing it, or are engaged in any of the arts that require their constantly inhaling its fumes, or handling it—gilders, for example—become pale, lose appetite, flesh and strength; the nervous system suffers in various ways; notably and most frequently, there is paralysis *agitans;* the bowels become loose, salivation, alopecia, pustular diseases of the skin, necrosis of the bones, etc., successively supervene, and, finally exhausted, death hurries off the victim to the grave. Mercurial cachexia is the name given to the above train of detailed symptoms. Its prolonged use is destructive to every form of animal and vegetable life; in sufficient dose some of its salts will kill either an elephant or a microbe.

### LOCAL ACTION.

The acid nitrate is destructive of tissue wherever applied. It is one of our best caustics. Corrosive sublimate, as its name indicates, corrodes and destroys animal tissues. It is escharotic, a corrosive poison; the best of all germacides, because it kills germs when more diluted or attenuated, with less constitutional and local disturbances, than any other agent of its kind, *e. g.*, carbolic acid, iodine, *et sui generis*. The red oxide is also escharotic.

Calomel, on the other hand, is sedative to ulcerated surfaces, causing healthy granulations to spring up, and thereby hastening cicatrization. It poisons, not by local, but by constitutional action. The larger the single dose the greater the immunity from harm With the corrosive salts this is reversed. The ointment applied to the skin is absorbed into the blood and produces characteristic symptoms. Frictions often occasion local troubles of the skin, especially on those who have a delicate cuticle, but it will do so on the toughest if the place where friction is practiced is not changed. Its fumes inhaled cause irritation of the mucous membranes lining the air passages, attended by cough and succeeded by systemic effects. Injected under the skin, no preparation yet used has been popular, or pleasant, on account of the irritation, tumefaction, pain, induration and frequently sloughing, which it is apt to occasion. Turpeth's mineral is an irritant emetic formerly much used in croup.

### CONSTITUTIONAL ACTION.

On the glandular apparatus. It is an universal stimulant to glandular secretion, both excretory and secretory. Not a gland in the body, probably, that is not stimulated by its presence. It is a poison, a something foreign to the economy, a substance which must be expelled. Calomel and blue mass act as purgatives by increasing the secretion from the liver, bile being the natural peristaltic stimulant; they also cause the pouring out of more of the pancreatic and other enteric juices. Now, it is a subject of controversy, and much has been said *pro* and *con*, as to whether mercurials do really increase the secretion from the liver. The results of the investigations of the Edinburg Commission caused some to doubt its action on the liver, others to concur with them fully in its non-action, among the latter the learned Stillé, of Philadelphia.

The remarks by Farquharson are so pertinent on this subject, that I read from his work to you what he says:

"The action of mercury on the liver has provoked a good deal of controversy, and whereas it was formerly held that the biliary secretion was directly stimulated, the experiments of Bennett, and the Edinburgh Committee seem to show that, on the contrary, the flow of bile is actually checked or diminished by calomel. Two obvious fallacies underlie these experiments, the first being that the dogs, kept for a considerable time previous, with biliary fistulæ, were so affected, not only by the shock of the operation, but by the resulting inconvenience, general discomfort and gradual starvation, that secretion must have been in a great measure suspended; and secondly, it is well known that a remedy which has no effect on a healthy organ may powerfully modify its condition when in a state of congestion or functional derangement." The National Dispensatory, one of the best books ever written, even with its abuse of mercury, says: "On the whole the most probable conclusion on this subject is expressed in these words: 'Calomel is not a cholagogue, but diminishes the secretion of the bile.'" The United States Dispensatory says: "As a purgative calomel owes its chief value to its tendency to work on the liver, the secretory function of which it stimulates." Again it says: "The alvine discharges, if clay-colored, are generally restored to their natural hue whether the liver be torpid and obstructed, as in jaundice, or pouring out a redundancy of morbid bile, as in melaena, the judicious use of mercury seems equally efficacious in unloading the viscus or restoring its secretion to a normal state." One of the most sensible men in England, Fothergill, in his Hand book of Treatment, says: "Mercury is a notable alterative. It is found in all excretions. It acts upon the flow of bile." Some say it acts upon the liver by irritating the ductus communis choledochus. It does not act this way, because it is not an irritant. It acts from its peculiar property, in secret and inexplicable. Water puts out fire, but it is made of one substance that is inflammable and another that supports combustion. The water has the inherent power to put out fire, and in the same way mercury has the power to act on the secretions. It has been proved that calomel does not act by irritating the intestines, nor alone by increasing the mucous secretion, but it acts as a cholagogue. If the bile fails to get in the duodenum the patient becomes yellow and is constipated. We know that mercury best restores the color and relieves.

The sensible rank and file of the profession never doubted sufficiently, at least, the cholagogue action of calomel to abandon its use, when they wished to act on this viscus. I say it stimulates all the glands to increased action. It is not a stimulant in the sense that alcohol or ether is. It is not a diuretic; but squills and digitalis may often be given for their action on the kidneys without increasing the urinary secretion; but the desired result will be almost certainly occasioned if a mercurial is added to them.

Of all the emmenagogues—an unreliable class I admit—the best I know of is Fenner's Tinct. Antacrid, which has in every ordinary dose about an eighth of a grain of corrosive sublimate. Mercury is not a diaphoretic, but arsenic cures a number of skin diseases of the squamous variety, attended by dry skin, but the best preparation of it to use is the liquor hydg. iodidii et arsenitis. Does mercury act on the pancreas? This secretion is essential for the emulsion of fats before they are absorbed. Cod liver oil will frequently disagree with your patient, it will not be digested, therefore do him no good. A dose of calomel, one to two grains at bed time, if he be scrofulous or tubercular it makes no difference, will quickly rectify this. It is no longer belched up, the appetite and flesh improves; it is digested. Such assimilation could not take place without pancreatic juice. We must credit the mercurial for its reappearance. The late Joseph Pancoast taught me that small doses of blue mass and calomel were, by insuring proper secretions and digestion, *tonics*, even in wasting diseases.

The salivary secretion is increased by mercury, so much so that salivation is our dread. Salivation is *toxic* effect, not *therapeutic* result. It is an effort of nature to rid the system of the poison. When the interdental spaces begin to swell, when there is a coppery taste in the mouth, a little soreness in the articulation of the jaws, or tenderness of the teeth when struck together, you have given enough mercury. Here medicinal action ceases and poisonous results begin.

Its protracted use deranges the digestion, and affects the nervous system in a manner to which sufficient allusion has been made.

It disorganizes the blood, lessening the number of red blood corpuscles, destroying its fibrin, impairing its plasticity, diminishing its salts and albumen, lessening its coagulability, and hastening, when drawn, its decomposition.

It is found in all the secretions and excretions from the body, and its presence has been detected in all the organs and tissues, bony, muscular, cellular, etc. It causes intractable ulcerations of the skin and mucous membrane of the mouth, periostial pains and even, it is said, nodes on the bones—the hair falls and the nails are sometimes shed. The resemblance to syphilis is so marked as to strike even the most casual observer. The anemia, which is a result of the blood dyscrasia, predisposes to hemorrhages.

Weeks after a profuse salivation it is still detected in the excretions. It is sometimes deposited in the tissues and months afterwards, being freed by the exhibition of iodide of potash it has caused ptyalism. A patient once salivated is more easily ptyalized a second time be there even an interval of years between them; hence I would advise you to always ask your patients when you give them calomel the first time if they ever suffered from its poisonous effects. Children and infants exhibit marked immunity from ptyalism, but remember you may not salivate the child, but worse than that sloughing of the buccal mucous membrane is often occasioned and the cicatrices formed when these surfaces are healed has led to ancylosis of the maxillæ, necrosis of this bone, etc.

Old cicatrices are often reabsorbed in those under the influence of this metal, and fibrinous exudations disappear. The impoverished state of the blood predispose to hydrops. The power of resisting cold is lessened, and the predisposition to contract all manner of disease increases. Is it any wonder that anti-mercurial schools arose, when it was once the orthodox fashion of treatment to salivate for almost every disease that flesh was heir to?

THERAPEUTIC ACTION.

So much for its physiological effects, especially when pushed to full action, but now we come to its therapeutic results, when rationally administered. In order to illustrate and keep constantly before you its properties, I have written on the black-board its virtues:

Alterative or catalytic.
Anti-syphilitic.
Tonic.
Anti-phlogistic.
Stimulant.
Purgative (cholagogue.)
Sedative.
Anthelmintic.
Germacide.

Alteratives are said to produce a salutary change in disease without sensible evacuation; catalytics destroy or counteract morbid materials in the circulating fluid. These definitions make mercury applicable in the treatment of all diseases. (?)

I know of many remedies for syphilis, but am acquainted with but one single agent that cures it, that is an antidote to the syphilitic virus, and that agent is mercury. When I say mercury is the cure for syphilis, I mean what I say, all that I say, and only what is said. Now venerial sores and chancroids are not syphilis, and for them mercury is to be eschewed. I have dealt with disease as something that enters the blood, an individuality, so to speak. Sigmond, the great Vienna syphilographer, says syphilis is dependent on a germ. Such has not been the teaching you have received from some of the older professors here. Some tell you that disease is perverted function, a lesion of enervation; and so it is, but I go a little further and ask what causes the perversion, what induces the lesion? A virus acts in an inconceivably small quantity and reproduces itself indefinitely. It is a living something. The virus from a chancre will reproduce its kind. That there will be discovered a corpuscle, or bacillus syphilitica, I have no doubt. The syphilitic virus is killed by mercury, (not only by the corr. sub. when actually applied to the virus, for acids and heat and caustics of all kinds will render the virus incapable of innoculation,) but the two will not inhabit the same fluid or tissues. In other words, a man under the medicinal influence, (and by this I mean with just enough mercury in him to be apparent, never toxically, that is, salivated, etc.,) of mercury for a protracted period, the germs of syphilis will die, they cannot and will not circulate together. Please bear in mind the close resemblance between the mercurial cachexia and syphilis, or else you may substitute for the latter, a disease every whit as fatal and incurable. Mercury is the antidote for syphilis, *primary*, *secondary* and *tertiary*, as they are conveniently classed, and on *clinical grounds*.

This being my position, of course, after I am *fully satisfied* that I have a hard chancre, *chancre*, to deal with, I do not delay a moment to put my patient on some preparation of the God-like metal. I assure myself that I am *correct* in my diagnosis, and then like Davy Crocket, "go ahead." I know this is not the orthodox teaching, but if my house was on fire, even had I built it fire-proof, I should not trust to the flames going out; I should not fear damaging the furniture with water because of this hope. I should begin to extinguish before the flames broke out from the roof. So I think would every sensible man.

Why not treat disease sensibly, act by the body as we would by the house. No, say some of the modern teachers, there are a certain per cent. of persons who will escape secondary symptoms, and in the same breath they say it is constitutional from the time the chancre appeared. This is a contradiction. Another objection is, if you were mistaken, the mercury has done the constitution irreparable harm. There is sense in this caution, especially where mercury is used to toxic effect. Others say you are inexcusable to begin the mercurial until secondary symptoms appear. Wait until the man is broken out, enveloped in flames, before beginning, you might injure his constitution; some furniture will be spoiled.

Again it is said the treatment masks and delays secondary manifestations. I believe it will, in a certain per cent., prevent them altogether and always mitigate its violence. My experience in the treatment of syphilis has been very great. I am sure I *generally* know chancre from chancroid, herpes, etc., when I see it, and carefully compare the initial lesion with the history of the case.

But I cannot warn you too much about the caution you should observe in believing patients' statements of the last time they had intercourse. Men honest and truthful about other matters, will prevaricate when it comes to giving testimony on these matters.

It is better to delay giving the mercurial at first, unless there be *no* doubt at all as to the nature of the infecting sore. Your books are very clear in their descriptions of the differential diagnosis between chancre, chancroid, herpes, etc.; but in practice you will find many, many cases that time and time alone will ellucidate. But on the first appearance of secondary symptoms, there can no longer be any room for hesitancy as to the course you should persue

I have for the past fifteen years used almost invariably this prescription in primary syphilis, after thoroughly destroying the sore, with nitric acid, or the acid nitrate of mercury. It was handed down to me by my fathers in medicine, Drs. H. G. Tate and A. W. Griggs:

℞.—Tinct Iodinii, . . . . . . ʒ iij.
Hydg. Corr. Chlo., . . . . gr. viii.
Spt. Frumenti, . . . . . . ℥ xii.

M ft. sol. Sig.—Teaspoonful after meals, with water.

Watch the gums, for you want medicinal action, *not toxic*.

If it disturb the bowels, add tinct. opii. This is kept up for from three to six months, and if no secondary symptoms appear lessen the dose of mercury, and substitute for the iodine, the iodide potash in from five to twenty grain doses, according to circumstances. Continue this for from three to six months. Then iodide potash alone, or with some tonic for six months after the last symptoms of the disease.

Patients will ask you naturally, how long will I have to continue medicine when they first apply to you for treatment; tell them for six months after you and he are sure he is well. He will be pretty sure to then ply you with this query: When will that be? Say to him God in Heaven only knows. The iodide of potash is a *remedy* for some of the manifestations of syphilis that has no equal. I would not be without it. But be not deceived, when you see a syphilitic node melt away before it like a snow bank before the summer's sun, into the erroneous conclusion that the patient is cured. That *symptom* of syphilis has been relieved—an ugly, jagged-edged, foul-conditioned ulcer under its healing power cicatrizes; but bear in mind, that you have only "filmed and skillmed the ulcerous sore: rank corruption mining all within still infects unseen."

Mercury, and it alone, so far as I know, can eradicate the disease. Opium will alleviate the pain of a malarial neuralgia, but quinine is the antidote to the poison. It has long been known to clinitians that under the beneficent influence of this drug, syphilitics gain flesh, color and strength; but it was only recently demonstrated by Dr. Keyes that the number of red blood corpuscles was actually increased in those suffering from syphilis, after they were brought under the gentle influence of mercury. Physiological experiments could never have demonstrated this fact. Mercury is not the only medicine that acts clinically in a manner totally at variance with physiological deduction. The prescription which I have given I do not insist on at all, so your compound contains mercury, that will be sufficient, and you may give it by the month, by inunction, hypodermically, or by inhalation. In the old tertiary cases, of course cod liver oil, food and regimen are essential. My first care, if the patient be so broken down as not to be able to take mercury, is to build him up so that he can take it, and you will be surprised often how much sooner he can bear it than you are aware. In this disease, as previously alluded to, in minute doses it is a tonic, hence probably always indicated, be the state of the system ever so low.

Now mercury will not cure every case of syphilis, neither is every case of any disease amenable to any treatment. Quinine is universally spoken of as a specific against the malarial poison. Yet people die every day from miasmatic emanations, quinine administered to the contrary notwithstanding.

Mercury is indicated in inflammation of all varieties. Inflammation causes constipation, lessens the excretion of the skin, kidneys, the tongue is foul, there is torpor and sluggishness, mental and corporeal, effete matters are retained in the blood, etc. This pathological process causes an increase of the fibrin of the blood. Mercury decreases that constituent. It is anti-phlogistic; it destroys that which inflammation creates; it puts to work the emunctories which the phlogosis has paralyzed. As an anti-inflammatory agent it may be thus compared with veratrum or antimony and blood-letting. The immediate effect of bleeding is mechanical; that of veratrum or antimony, nervous; that of mercury, hæmetic.

Blood-letting weakens the force of the heart by diminishing the pressure on the vessels; antimony or veratrum diminishes the pressure on the vessels by weakening the force of the heart, and mercury does both of these things by impoverishing the blood. Antimony and veratrum arrest inflammation by reducing the pulse; mercury reduces the pulse by arresting inflammation;" antimony or veratrum direct their powers to the effects; mercury to the cause.

This extraordinary power of the drug has led to its almost universal use, for there are few diseases in which there is freedom from inflammation. When we did not understand the natural course of diseases as we do now; when we were afraid that if left to itself it would never tend to recovery, blood-letting and mercury held full sway. We know better now, and have found out that the large majority of diseases with proper nursing, etc., run their course without fatal result. But we must not go too far in tentative treatment, we must do something more than make a correct diagnosis and prognosis. Mercury is a stimulant to the glandular organs, excretory and secretory. Nature, always conservative, is helped by this drug in her effort to rid the system of the *materies morbi.*

Calomel is one of the very best of purgatives, but you should always see that it acts on the bowels; if after eight or ten hours catharsis is not occasioned by a full dose, give a saline or castor oil. In beginning the treatment of dysentery it is especially indicated. In hemorrhoids, especially those brought on, as is so often the case, by constipation, a mercurial is a very necessary ingredient in the laxative that you prescribe—for this anatomical reason—the hemorrhoidal tumors when recent are the results of congestion in the hemorrhoidal plexus of veins; these veins empty into the messenteric; these into the portal vein, the latter vein as you know ramifies in the substance of the liver as an artery, and in this gland the dam is found. Mercury by relieving this congested organ, permits of the disgorgement in the far off rectal vessels.

At one time mercury in conjunction with opium was regarded as essential in the treatment of serous inflammations, especially peritonitis, but it has been shown that the opium and not mercury is the curative agent. In syphilitic iritis, of course belladonna is indispensable, but if there be a disease in which you are excusable for salivating it is in this. In acute inflammations of the liver, I would not give mercury, on the principle of not working a sick horse. Calomel in small or large dose has been for time immemorial used as a sedative to the stomach in obstinate vomiting. I would not recommend it until other means had been exhausted, for these reasons: Nutrition is already impaired by the starvation which the non-retention of food has occasioned; except in syphilis it is not tonic, and never a reconstituent one; and again, if it did not purge I should fear toxic effect; and purgation would but increase the debility and weakness which is the natural result of this derangement of digestion. The best of all anthelmintics is calomel and santonine, followed by oil and turpentine. The santonine may often be left out, and still the parasites are expelled. As a purgative for children calomel has no equal in the long list of remedies of this class.

Few Southern doctors have written text books; the Northern and European writers tell you that a mercurial purge in the treatment of malarial diseases is unnecessary. Salines do just as well, say they, followed by quinine. They may in New York or Philadelphia, but you will find that if you do not "prepare the system" for quinine with calomel, the exacerbations will recur with provoking and mortifying frequency. The "preparation" which the mercurial gives I believe to be this, it insures the *absorption* of the anti-periodic, for unless it gets into the blood the *palmellæ* which cause the congestions, etc., are not killed. Fothergil in his Handbook of Treatment makes what I consider the following sensible remarks: * * "In convalescence the occasional use of alteratives is proper and beneficial. It often happens that a steadily progressive recovery is suddenly clouded by a state of feverishness, a foul tongue, loss of appetite and general *malaise.* Under these circumstances it is a good plan to give some pil. calomel, et colicinth co. at bedtime, and some citrate magnesia in the morning, or to give a few grains of calomel with some jalap or scammony in the morning if the patient be seen in forenoon. A gentle action on the bowels generally restores the condition to what is to be desired. But it must not then be conjectured that it is the mere purgative action which is the whole matter; like results will not follow if the *mercurial* be omitted."

We come now to its use in diphtheria. In fourteen years of practicing medicine I have not seen many cases of diphtheria. I did not call all sore throats or membranous deposits diphtheria. I would advise you not to do so. You will get notoriety but not reputation. You will become notorious among the people in a small area for curing diphtheria, but physicians will know you are an ignoramus, if you say you cure every case of diphtheria. Every case I had in these fourteen years died. I doctored them *secundum artem*, exactly as the books said, iron, stimulants and chlorate of potash. They all died scientifically. Mercury was withheld as a poison. I had been taught that the disease began with debility, and that the doctor who would give mercury was worse than a heathen. I concluded not to follow longer this treatment. I would advise you not to follow the lead of him who carries you to disaster. I remembered seeing an article in the London *Lancet* in 1871, which told of twenty cases of diphtheria cured by calomel and the bicarbonate of soda. I was disposed to ridicule it at the time. There was an article read before the American Medical Association upon corrosive sublimate in the treatment of diphtheria by Dr. Pepper. The writer details a case that was given large quantities hypodermically. The case was *in articulo mortis.* It got well. It was the only case he had. Dr. Gray and myself had a case of diphtheria together 2 years ago. Though the patient was nearly gone we were afraid of the mercury treatment. We knew of it and spoke of it. We treated the case secundum artem. It died. There was another child with diphtheria to which we were called. We gave it two grains of calomel every two hours. For a while it did not act. But in thirty-six hours there was a tarry discharge. We gave that child as much as fifty grains of calomel. It got well. We kept up the stimulants, milk, and punches all the time. In the same family in a very short time there was another child taken. It was treated like the last, it was given 100 grains; it recovered. Some of the best physicians in the city said we were mistaken in our diagnosis, that if there had been diphtheria mercury would have killed them. They said if this had been true diphtheria there would follow the sequale, albuminuria, aphonia, etc. This case had all of these. I have treated six cases in the past two years in this way, and five have recovered. I expect to continue this treatment until I can find something better. Hereafter I believe I will use locally a solution of the bichloride, two grains to the ounce of water, and paint it on the tonsils and pharynx with a camel's hair brush. If it is true that germs cause the disease, this application will kill them. It may be that from these deposits on the tonsils and the pharynx and all in the throat, these germs drop down and the patient swallows them. They are then absorbed and carried over the whole economy to be deposited wherever there is an abraded surface. Since these articles have been written much has been said on this subject. If this, the germ theory of diphtheria, is not true, another reason for its use is the formation of heart clot, which is one of the greatest dangers in this disease. The heart is stopped. Mercury defibrinates the blood, and without fibrine these clots could not be. We thus avoid one of the great and dangerous results of diphtheria; and lastly, another reason why I approve the treatment, and it is one of the very best of reasons, under the approved mode of medication, my patients died, under mercury they recover. No case was salivated. You will please observe that the principle of treatment is the same, alcohol and the chlorides are both germicides, so is mercury. In the treatment of diphtheria with mercury I object to such tremendous large doses as 20-40 grains, because we do not have the signal of danger; it does not salivate. Small doses, 2 or 3 grains every two or three hours is sufficient. Put it down as a golden rule, learn it by heart, and don't forget it, that ptyalism is not a therapeutic result but a toxic effect.

Typhoid fever is treated by the Germans on what they call the eliminative plan during its first week. It is very often exceedingly difficult to tell for a week or ten days whether the fever you have to deal with be typhoid or not. Their plan is to give ten grains of calomel every other morning for a week. I am not prepared to indorse this plan fully, but the following good results that would accrue, recommend it. The large dose insures purgation, which so early in the case will do no harm, so ptyalism is obviated. There is always an effort on the part of nature to throw off disease, calomel stimulates all the emunctories. If the disease be caused by germs it is emphatically germicide. I dare not deny that it occasionally aborts it, though I cannot say we have any specific as yet for the typhoid poison; in malarial localities the calomel and quinine at first are due the patient. Often if they are omitted your patient will go into a typhoid condition, when at first he, by their judicious administration, would have recovered in a few days, and been saved weeks of suffering and perhaps death.

I have found that unless I gave calomel after relieving a child of ordinary croup with emetics, the disease was very apt to recur for three nights in succession.

Corrosive sublimate, one to two parts to the thousand, is rapidly taking the place of other antiseptics. Lister has recently sanctioned its use in the making of his antiseptic gause; Thomas, of New York, uses it for disinfecting his instruments, hands, etc., and as an antiseptic wash in surgical operations and as a preventive of puerperal fever.

Locally, calomel is used in various diseases, so is the ammo. precip. hydg., red oxide and corr. sub., which it is not necessary for me to enumerate in this place, although it was fully entered into before the class. Neither do I deem it profitable or interesting to my readers to mention all the diseases in which we use mercury, its modes of administration, etc. The following prescription I have found so useful in parasitic diseases of the skin that I give it: ℞—Hydg. Corr. Chlo grs. iv, Tr. Iodinii, Tr. Canthar. Spt. Vin. aa. q. s. ad. ℥i, M. ft. sol. Sig. Paint on with a camel's hair pencil. Pruritis vulvæ and anni are often occasioned by parisites, Corr. Sub. 2 grs. to the oz. of alcohol killing them, the itching ceases.

In conclusion I desire to make this remark: Three of our most used and most highly valued medicines are germicides, viz: alcohol, quinine and mercury.

We are on the eve of great discoveries; a bright day is dawning upon us, after the long night of doubt and uncertainty about the *modus operandi* of drugs, a great flood of light is being shed on this subject by such men as Pasteur and Koch. I verily believe that the time will come when, owing to the advances in the healing art, the average age of man will be three-score years and ten, and lo! the day is near at hand!

